# Routine Whole-Genome Sequencing of Hospital Bacterial Isolates Identifies the ED as a Source of Transmission

**DOI:** 10.1017/ash.2025.373

**Published:** 2025-09-24

**Authors:** Anastasia Weiland, Jennifer Yim, Kathleen Quan, Cassiana E. Bittencourt, Keith Madey, Alison Gassett, Emma Briars, Mohamad Sater, Nicole Billings, Julie Shimabukuro, Neil Uy, Thomas Tjoa, Susan Huang

**Affiliations:** 1 Division of Infectious Diseases, University of California Irvine School of Medicine; 2 University of California Irvine Health; 3 University of California, Irvine; 4 UCI Medical Center; 5 Day Zero Diagnostics; 6 University of California Irvine School of Medicine

## Abstract

**Background:** Hospital outbreak detection depends on microbial cultures that cluster in time and space, generally defined as inpatient units. Data are lacking on whether Emergency Departments (EDs) may be a source of transmission. **Objective:** Assess ED exposure as a source of transmission using routine whole-genome sequencing (WGS) of bacteria from ED and inpatient specimens in a tertiary academic medical center. **Methods:** We performed a prospective cohort study of patients at a 450-bed academic medical center who had bacteria isolated from ED and inpatient specimens between April 1, 2022 and March 31, 2023. Each organism per patient specimen was routinely sent for WGS and genomic clusters were identified as two or more bacterial isolates cultured from different patients which were genomically related by WGS, generally, 25 or fewer single nucleotide polymorphisms apart. Retrospective chart review using a standardized assessment form was conducted for patients involved in hospital-onset genomic clusters to assess for epidemiologic links occurring in the ED versus other hospital areas (inpatient or outpatient). **Results:** During a 1-year period, 3614 isolates were sent for WGS with 44 genomic clusters identified. Thirty (68%) clusters were excluded because they consisted of community-onset cases, suggesting either transmission outside of our hospital or acquisition of a common community strain. Fourteen hospital-onset clusters were evaluated for possible ED transmission. Of the 14 clusters, median cluster size was 2 patients (range: 2-5). Most common pathogens were Enterococcus faecalis (N=4), Pseudomonas aeruginosa (N=3), and Staphylococcus aureus (N=2), with more Gram-positive (N=8) than Gram-negative (N=6) clusters. Nine (64%) clusters had evidence for ED transmission, which were categorized as probable (ED as sole opportunity for exposure, N=5), or possible (ED among multiple healthcare opportunities for exposure, N=4). Examples of ED exposures included (non-mutually exclusive): same ED unit (N=9, 100%), proximal time in ED (≤5 days apart), (N=6, 67%), common ED staff (N=5, 56%). Without WGS, identification of the ED as the probable source of transmission was hampered by the fact that 99% of cultures were taken on different units, with a median time between ED exposure and positive culture of 9 days (range: 0-163). **Conclusions:** Routine WGS of bacterial isolates in a 450-bed tertiary care center identified 14 hospital-associated transmission events in a year, two-thirds of which were probably or possibly associated with the ED. ED transmission is common but difficult to identify because infection prevention processes rely on positive cultures collected in the same unit close in time.

